# Nephrologists’ perspectives on dialysis treatment: results of an international survey

**DOI:** 10.1186/1471-2369-15-16

**Published:** 2014-01-15

**Authors:** Richard J Fluck, Denis Fouque, Robert S Lockridge

**Affiliations:** 1 Department of Nephrology, Royal Derby Hospital, Derby, UK; 2 Department of Nephrology, Hôpital Edouard Herriot, Lyon, France; 3 Division of Nephrology, University of Virginia Health System, Charlottesville, VA, USA

**Keywords:** End-stage renal disease, In-centre haemodialysis, Home-based haemodialysis, Practitioner attitudes, High-dose haemodialysis

## Abstract

**Background:**

In-centre haemodialysis (ICHD) is the most common dialysis method used by patients worldwide. However, quality of life and clinical outcomes in patients treated via ICHD have not improved for some time. ‘High-dose’ haemodialysis (HD) regimens – which are longer and/or more frequent than conventional regimens and are particularly suitable to delivery in the home – may offer a route to improved outcomes and quality of life. This survey aimed to determine nephrologists’ views on the validity of alternatives to ICHD, particularly home HD and high-dose HD.

**Methods:**

A total of 1,500 nephrologists from Europe, Canada and the United States were asked to respond to an online questionnaire that was designed following previous qualitative research. Certified nephrologists in practice for 2–35 years who managed >25 adult dialysis patients were eligible to take part.

**Results:**

A total of 324 nephrologists completed the survey. ICHD was the most common type of dialysis used by respondents’ current patients (90%), followed by peritoneal dialysis (8%) and home HD (2%). The majority of respondents believed that: home HD provides better quality of life; increasing the frequency of dialysis beyond three times per week significantly improves clinical outcomes; and longer dialysis sessions performed nocturnally would result in significantly better clinical outcomes than traditional ICHD.

**Conclusions:**

Survey results indicated that many nephrologists believe that home HD and high-dose HD are better for the patient. However, the majority of their patients were using ICHD. Education, training and support on alternative dialysis regimens are needed.

## Background

End-stage renal disease (ESRD) places a considerable burden on healthcare resources [1]. Over 2 million people worldwide currently require treatment for ESRD [2]. In 2009, over 1 million patients received renal replacement therapy (RRT) in the USA and Europe combined [3,4]. Despite technological advances in haemodialysis (HD) over the past 2 decades, clinical outcomes remain poor and high rates of morbidity and mortality persist [5]. In the USA, HD patient survival rates have barely improved in more than 25 years and, in 2009, only 50% of dialysis patients were expected to survive 3 years after the start of ESRD therapy [3]. In addition to the lack of progress in survival rates, the quality of life of patients on HD has not improved [6].

In-centre HD (ICHD), generally performed thrice-weekly for 4 to 5 hours per session, is the most common dialysis option used by patients worldwide [3]. However, the latest USRDS report shows that 33.6% of HD patients in the US receive only 180 minutes of dialysis or less per session [7]. Alternative, home-based dialysis options include peritoneal dialysis (PD) and home HD. While not true for all patients [8-10], generally, PD and conventional home HD offer clinical outcomes that are equivalent, and in some patient groups superior, to those provided by ICHD [11-15]. However, patients using home dialysis benefit from greater independence and autonomy, and less intrusion into their everyday lives [13,16,17]. Furthermore, reduced travel, support, service and collateral costs mean that home dialysis can produce equivalent or greater clinical benefit at reasonable or even reduced costs [12,18]. Home HD has the added benefit of facilitating frequent and/or longer ‘high-dose’ HD regimens such as nocturnal HD and short-daily HD. Such regimens lack the long (2-day) interdialytic interval associated with conventional HD, which has been shown to increase patient mortality risk [19]. In contrast, high-dose HD regimens have shown significant physiological, clinical and patient-reported advantages over conventional ICHD [20-30]. Despite their apparent benefits, home dialysis options remain the less popular choices in many countries [3]. In light of the apparent disconnect between the possible advantages and actual uptake of home dialysis, a multinational survey of nephrologists was undertaken to determine nephrologists’ views on home dialysis options, with a particular focus on home HD and high-dose HD.

## Methods

Nephrologists from Canada, France, Germany, the United Kingdom and the United States were asked to respond to an online questionnaire that took around 45 minutes to complete. The invitation to participate was sent electronically to a list of 1,500 known nephrologists within the participating countries. Questionnaires developed in English and translated into appropriate languages for nephrologists in France and Germany were completed by respondents in the second half of 2010. Respondents were paid a fee to complete the questionnaire in line with currently acceptable standards for such research. The first part of the questionnaire contained questions used to determine whether the responder was eligible to take part in the survey. Only certified nephrologists who had been in practice for 2–35 years and who currently managed more than 25 adult dialysis patients on any modality were eligible for inclusion in the survey analysis. Responders were not included if they currently served as a consultant, advisory board member or employee of a pharmaceutical company, medical device company or other healthcare manufacturer; or had participated in dialysis market research within the past month.

The second part of the questionnaire was designed to obtain nephrologists’ beliefs and attitudes around dialysis modalities and prescriptions, and current treatment goals. Questions were chosen based on previous, unpublished qualitative research performed in the five aforementioned countries. The qualitative research explored the beliefs and values of nephrologists and nurses towards dialysis in general and home dialysis options in particular, and was performed using a variety of research techniques, including in-person, in-depth interviews and in-depth interviews by telephone. Interviews lasted from 45–120 minutes and provided directional information to structure the quantitative questionnaire.

Nephrologists were asked to respond to a variety of statements using the following scale: strongly disagree; disagree; somewhat disagree; somewhat agree; agree; strongly agree. Specifically, questions were asked to discover nephrologists’ personal motivations for treating chronic kidney disease or dialysis patients, treatment goals, attitudes towards new therapies, attitudes towards guidelines and policies, attitudes towards dialysis modalities, and reasons for selecting particular dialysis modalities. In the third section of the questionnaire, the physicians were asked to summarise the dialysis modalities used by their patients, and also general patient capabilities and health profiles. In the final section, different patient profiles were described (relatively healthy; moderately healthy; chronically unhealthy) and nephrologists were asked to provide their dialysis modality recommendations for each profile. This was an extensive survey and the questions most relevant to physicians’ dialysis modality selection are reported herein (see Additional file 1).

Results were analysed using Excel and presented as descriptive statistics. Data are presented as mean numbers ± standard deviation, or as percentages. Unless otherwise stated, all respondents who indicated ‘strongly agreed’ or ‘agreed’ are presented together in the text as the proportion of respondents who agreed with a particular statement.

Ethics approval was not sought for this online survey of healthcare staff; consent to participate in the survey was considered to be implied by a response from the participant.

## Results

### Respondent characteristics

A total of 324 nephrologists from five countries completed the survey; almost half of all participants came from the USA (Table 1). On average, clinicians spent 57.2% of their time working in hospitals (ranging from 19.5% in Germany to 98.4% in the UK) and 34.3% of their time in private practice (ranging from 0.5% in the UK to 65.3% in Germany). Mean values for the estimated number of adult dialysis patients currently treated (per respondent) ranged from 97.1 (±48.2) in France to 342.7 (±208.5) in the UK (Table 1). Overall, participants had a mean of 14.1 ± 7.5 years of nephrology practice (excluding time as a trainee). Approximately 20% of patients currently attending responders’ clinics had started dialysis in the last year.

**Table 1:** Number of participating nephrologists and mean number of dialysis patients per respondent by country

	**Number (%) of participating nephrologists**	**Mean (± SD) number of adult dialysis patients treated per nephrologist**
Canada	46 (14.2)	168.0 (118.3)
France	47 (14.5)	97.1 (48.2)
Germany	31 (9.6)	109.0 (51.2)
UK	50 (15.4)	342.7 (208.5)
USA	150 (46.3)	128.4 (109.7)

*SD*, Standard deviation.

### Characteristics of dialysis prescription by nephrologists

**Figure 1:**
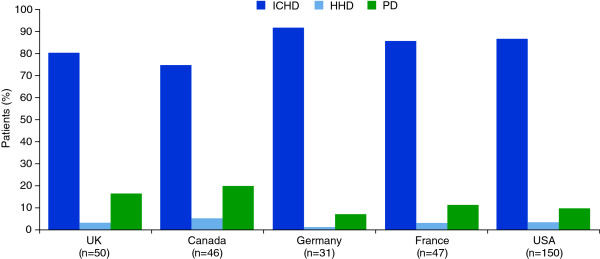
**Proportion of patients receiving different forms of dialysis treatment by country.** HHD, home haemodialysis; ICHD, in-centre haemodialysis; PD, peritoneal dialysis, n = number of respondents per country.

ICHD was the most common type of dialysis prescribed. Overall, nephrologists reported that 90% of their patients were on ICHD; PD (8%) and home HD (2%) were much less commonly used. This predominance of ICHD was observed across all five countries (Figure 1). The proportions of nephrologists who reported that their dialysis clinic did not offer home HD varied from 8% in the UK to 36% in France, and the proportions of nephrologists reporting that their clinic did not offer PD varied between 4% (UK and USA) and 19% (Germany) (Table 2).

**Table 2:** Home HD and PD availability across each country

	**Home HD not offered in dialysis clinic/unit (% [n])**	**PD not offered in dialysis clinic/unit (% [n])**
Canada	13 (6/46)	9 (4/46)
France	36 (17/47)	13 (6/47)
Germany	23 (7/31)	19 (6/31)
UK	8 (4/50)	4 (2/50)
USA	18 (27/150)	4 (6/150)

### Nephrologists’ attitudes to treatment goals and dialysis prescribing

**Figure 2:**
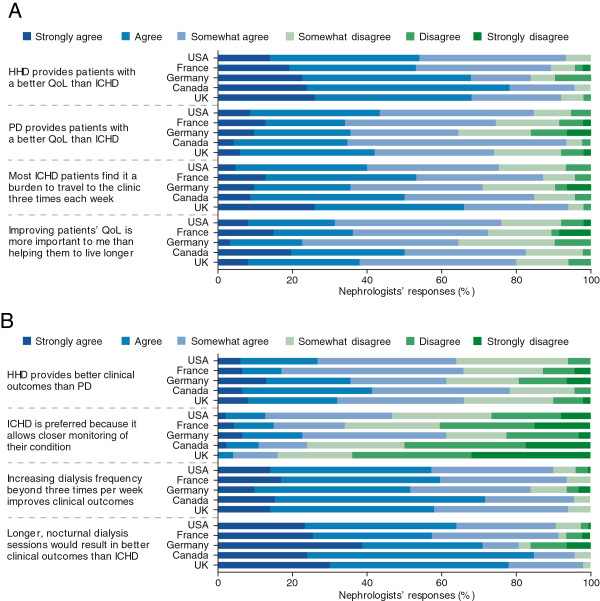
**Nephrologists’ attitudes towards different dialysis modalities.** Responses to statements around the effects of different dialysis modalities on **(A)** quality of life and **(B)** clinical outcomes. HHD, home haemodialysis; QoL, quality of life; ICHD, in-centre haemodialysis; PD, peritoneal dialysis.

Overall, 35% of nephrologists agreed or strongly agreed with the statement that “improving patients’ quality of life is more important to me than helping them live longer”. When nephrologists who stated they somewhat agreed with this statement were included, 76% of nephrologists were shown to favour quality of life improvements versus increased survival (Figure 2A). Approximately 61% of nephrologists agreed that home HD gives a better quality of life than ICHD and 47% agreed that ICHD patients find it a burden to travel to the clinic three times each week (Figure 2A). The majority also agreed that increasing the frequency of dialysis beyond three times per week significantly improves clinical outcomes (59%), and that longer dialysis sessions performed nocturnally would result in significantly better clinical outcomes than traditional ICHD (69%) (Figure 2B). Only 12% of nephrologists agreed that they preferred their patients to have ICHD because it allowed closer monitoring of their condition (Figure 2B). A total of 61% of respondents thought that home HD was under-prescribed and 62% thought that PD was under-prescribed. Only 8% of responders agreed that they needed to see more clinical data before prescribing home HD. Overall, nephrologists’ attitudes towards different dialysis modalities were similar across all five countries (Figure 2A and B).

### Nephrologists’ recommendations on dialysis

Patient health was important in treatment recommendation in all countries. Over half of nephrologists (56%) agreed to some extent (strongly agree; agree; somewhat agree) that they would recommend home HD or PD only to their healthiest patients. Family and home status was also an important factor; 45% of nephrologists agreed that patients must have a care partner before they would prescribe home HD. Respondents estimated that as many as 35% of their patients had no care partner.

**Figure 3:**
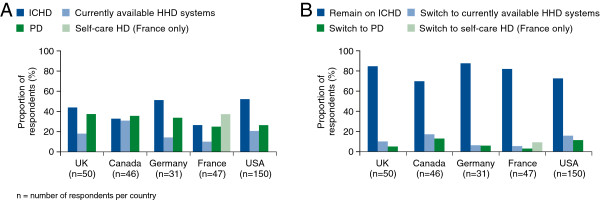
**Nephrologists’ dialysis recommendations to patients in fictional case studies. (A)** Patient has relatively good health and is new to dialysis; **(B)** Patient has relatively poor health and is already on ICHD. HHD, home haemodialysis; ICHD, in-centre haemodialysis; PD, peritoneal dialysis. *Self-care HD = patients attend outpatient facilities with no/very limited nurse supervision and no attending doctor (France only).

Half the nephrologists (50%) stated that home HD was their preferred treatment option and 63% said they would recommend it to the majority of their close family or friends. In contrast to this, use of a range of fictional patient case studies showed that the greater proportion of respondents in all countries except Canada would recommend ICHD rather than home HD to a patient who was relatively healthy and new to dialysis (Figure 3A). When presented with a fictional patient in relatively poor health already on ICHD, the majority of responders in all countries stated that they would expect the patient to still be on ICHD in one year’s time (Figure 3B).

## Discussion

A recent paper has highlighted four areas of benefit to patients from high-dose HD: 1. improved physical and mental wellbeing, 2. increased control over their life, 3. decreased sick role (including return to regular employment) and 4. identification of competencies to undertake self-care [31]. In support of this, our study showed that most participating nephrologists would prefer to prescribe home dialysis. Most nephrologists who responded to the survey believed that home HD provides a better quality of life than ICHD. In addition, most stated that they would recommend home HD as a dialysis treatment to their family and friends, and felt that home HD, as well as PD, were under-prescribed. Despite such apparently overwhelming support for home dialysis from respondents, this survey also showed that the vast majority of dialysis patients under respondents’ care currently receive ICHD. Furthermore, investigation into which dialysis modality respondents would recommend for patients with different health profiles revealed that, despite preferring home dialysis, most nephrologists in France, Germany, the UK and the USA would still recommend ICHD to patients, regardless of health status or history of dialysis. However, it was noted that a slightly greater proportion of respondents would recommend home HD and PD if the patient was relatively healthy and new to dialysis. Over half of respondents agreed to some extent that they would recommend home HD or PD only to their healthiest patients.

The majority of respondents considered that increasing the frequency of dialysis beyond three times per week and performing longer nocturnal dialysis sessions significantly improves clinical outcomes. These beliefs are consistent with evidence from clinical studies indicating that high-dose HD, i.e. short-daily or nocturnal HD, can improve physiological markers and clinical and patient-reported outcomes versus conventional ICHD. For example, patients on high-dose HD regimens exhibit improved urea clearance [20-22], lower phosphate levels and use of phosphate-binding medications [22,25,26], greater blood pressure control [22-24] and reduced left ventricular mass [24,25] versus those on ICHD. Patients receiving high-dose HD also report an improved perception of their general health and mental health and a reduction in the impact of disease on their lives [27-30]. Survival data are relatively scarce, but two small retrospective, observational studies have reported better survival for patients on nocturnal HD versus those on ICHD [32,33]. A retrospective matched-cohort study also reported a 45% improvement in survival with high-dose HD in the home versus thrice-weekly ICHD [34]. Daily home HD has also been associated with a 13% lower risk for all-cause mortality than conventional ICHD performed three times weekly [35].

Two main questions arise following our survey: why is there a large discrepancy between nephrologists’ treatment preferences and their prescribing habits, and what can be done to reduce it? One likely factor is treatment availability; for example, up to a third of the respondents’ dialysis clinics did not offer home HD, although this reduced to up to a sixth of respondents for PD. In the surveyed countries, ICHD is the standard treatment option, and it may be more difficult for a clinician to prescribe an alternative. One study showed that the use of home HD in the USA was influenced by the number of treatments covered by Medicare [36]. In an Australian study, lack of physical clinic infrastructure and training facilities were among the reasons cited by nephrologists as barriers to the uptake of home HD [37]. In some countries PD is the standard method of dialysis and infrastructure is well developed to support this. Therefore, the discrepancy between nephrologists’ home dialysis preferences and prescribing practices in these countries may be less pronounced [17].

Our results show there appears to be a perception among the respondents that home HD and PD should be used only in patients who are relatively young and healthy. Comorbid conditions can influence the best type of dialysis modality to use. For example, if a patient has multiple comorbidities or suffers from frequent complications during HD, then ICHD under medical supervision may be necessary [17]. Similarly, patients with diabetes or comorbid heart conditions are more likely to use PD than home HD [38]. However, it is not always the case that healthier, younger patients are best suited to home dialysis; with adequate support, older patients with comorbidities can fare well on home HD or PD [39]. Studies further profiling which patients benefit most from home HD may help nephrologists when recommending different dialysis modalities to their patients.

Several studies have been published investigating healthcare provider and patient attitudes towards different methods of HD care and delivery, including home HD [37,40-42]. A report investigating home HD in the USA categorised the impediments to its effective delivery into educational barriers (for patients and healthcare providers), governmental/regulatory barriers (state and federal), and barriers specifically related to the philosophies and business practices of dialysis providers (e.g. staffing, supplies and continuous quality improvement practices) [43]. In Australia, nephrologists felt healthcare system factors such as inadequate funding for home therapies in the private sector, lack of financial incentives in the public sector, limited psychological outreach support for patients and carers, lack of training facilities and opportunities for staff and patients, and lack of available simple home dialysis technology prevented the increased uptake of home HD [37]. Nephrologists and nurses believed that patients might also be worried about the personal costs of home HD, even though many costs (such as the financial burden of travelling to a clinic) are actually reduced with home HD [12,17,37,40]. Patient attitude and lack of confidence and motivation were also cited as barriers, especially if patients had begun therapy with ICHD and had already been on dialysis for a long time [37,40].

In countries such as Australia, Finland and the UK where the uptake of home HD is relatively high, success has been attributed to provision of adequate funding, support, education and training to both service users and service providers in the use of home HD [17,44,45]. In Australia, nephrologists believed that medical and nursing expertise in home dialysis was good, that home HD was available and supported by most units, and that longer hours and/or more frequent regimens offered outcome advantages [37]. At the Helsinki University Hospital in Finland, adoption of a ‘home first’ policy in predialysis education, close cooperation with other dialysis centres and centralised home HD training to support remote hospitals were cited as key factors for establishing an effective home HD programme [44]. Strong clinical leadership appears to be key in the UK, with the need to challenge beliefs about who might be suitable for home HD emerging as a consistent theme for improving patient access to home HD [45]. Additional considerations for successful home HD programmes, raised by the American Society of Nephrology Dialysis Advisory Group, include: selection of appropriate dialysis machines for the treatment regimen, differences in prescribed regimens e.g. dialysate flow; reliable vascular access, preferably arteriovenous fistulas; the potential requirement for remote monitoring, and finally, patient burnout necessitating return to ICHD or a period of ‘respite’ care [46]. Further initiatives to establish integrated home HD pathways, develop financial incentives and solutions to sustainability challenges, provide support for carers, and capture key indicators of dialysis use and practices within renal registries are also required [18,45]. Initiatives such as telemedicine (i.e. telephone support lines and other information technology applications) to provide medical support for patients at home could help improve safety and reassure patients on home HD [42].

The results of this survey must be interpreted in light of its limitations. Generally speaking, a survey can capture only broad perspectives. Views on complex issues are difficult to compress into simple answers and the questionnaire could not capture all the nuances of nephrologists’ treatment decisions. In France, for example, funding for a nurse to provide assisted PD is available to patients, from which elderly patients who would otherwise be on ICHD can benefit. Therefore, this is likely to influence French nephrologists’ treatment recommendations. It should also be recognised that respondents’ answers to interview questions may differ from their true beliefs. In addition, although encompassing five countries, the study results were biased towards experiences of US nephrologists, as the number of USA respondents was around three times greater than for other countries. Outside the USA, only a relatively small number of respondents (≤50) were recruited from each country. It should also be noted that the survey did not include respondents from Australia and New Zealand, countries where home therapies are most common. Data from a larger, more comprehensive group of nephrologists, along with appropriate statistical analysis, would help further elucidate worldwide practices and preferences in dialysis treatment. Finally, the process of recruiting survey respondents may have itself biased the study population in some way, perhaps recruiting only the most enthusiastic professionals. Therefore, survey results may not fully portray the full spectrum of nephrologists’ attitudes. Of note, in another international survey comprising 544 respondents, physician attitudes toward the evidence for high-dose HD differed significantly between those who typically had patients on high-dose HD and conventional ICHD providers [47]. High-dose HD providers were significantly more likely to agree with statements that such regimens improve quality of life, improve nutritional status, reduce erythropoietin requirements and are cost effective compared with ICHD providers [47].

## Conclusions

In summary, this survey revealed a high level of support for home therapies, including home HD. Despite this, most patients under the respondents’ care are treated by ICHD, consistent with the generally low uptake of home HD in clinical practice. While lack of access may partially explain these low uptake rates, nephrologists may be excluding certain types of patients from home therapies (e.g. older patients, individuals with comorbidities) who could actually benefit from options other than ICHD. Provision of more support, training and education to nephrologists and other members of the multi-professional team is needed to increase clinicians’ knowledge of, and confidence in, the suitability of home HD for the wider patient population. Such training and education must be embedded into the professional curriculum and should encompass the relative merits of all modalities including the specifics of home therapies in both training and ongoing care. In centres not offering home therapies, trainees and established clinicians should be offered fellowships or other opportunities to learn about home treatments. Shared decision making between clinical teams and their patients should also be improved. In addition, new incentives to drive changes in national healthcare policies may be needed.

## Abbreviations

ESRD: End-stage renal disease; HD: Haemodialysis; HHD: Home haemodialysis; ICHD: In-centre haemodialysis; PD: Peritoneal dialysis; QoL: Quality of life; RRT: Renal replacement therapy; SD: Standard deviation.

## Competing interests

RJF has received lecture and consultancy fees from Baxter, Gambro, Amgen, Ortho Biotech and Roche. DF has received lecture fees from Amgen, Fresenius, Genzyme and Shire, and consulting fees from Abbott, Amgen, Baxter, Danone, Genzyme and Shire. RSL is a member of the Machine Medical Advisory Board of Fresenius Medical Care.

## Authors’ contributions

RJF, DF and RSL contributed to the analysis and interpretation of the data, drafting and revising the article, and provided intellectual input. All authors read and approved the final manuscript.

## Authors’ information

RJF is the Clinical Lead for the East Midlands Renal Network, Fellow of the Royal College of Physicians, and President of the British Renal Society (britishrenal.org). RSL is a Clinical Associate Professor of Nephrology, University of Virginia, Medical Director UVA Lynchburg Home Dialysis Program and one of the primary investigators for the FHN Nocturnal Trial.

## Pre-publication history

The pre-publication history for this paper can be accessed here:

http://www.biomedcentral.com/1471-2369/15/16/prepub

## Supplementary Material

Additional file 1 **Questionnaire.** Survey questions relevant to physicians’ dialysis modality selection. Click here for file
