# Supplementary material for: Nephrologists’ perspectives on dialysis treatment: results of an international survey
Source: BMC Nephrol. 2014 Jan 15;15:16. doi: 10.1186/1471-2369-15-16 (PMC3912927; doi:10.1186/1471-2369-15-16)
Supplement: Additional file 1 — Questionnaire. Survey questions relevant to physicians’ dialysis modality selection. [file 1471-2369-15-16-S1.pdf]

Questionnaire

Thank you for agreeing to participate in this important research.

Your feedback and opinions are very important to us. As a token of our appreciation for your time, we will be offering an honorarium to each physician who qualifies for the study and who completes the survey.

This survey will take approximately 45 minutes of your time.

Please be assured that all information you provide will be kept strictly confidential. Study sponsors will NOT have access to your name, address or phone number. Please note that the information discussed is for research purposes only.

Screener

1. What is your primary medical specialty?
   - ☐ Allergy
   - ☐ Cardiology
   - ☐ Endocrinology
   - ☐ Gastroenterology
   - ☐ Nephrology
   - ☐ Surgery
   - ☐ Other specialty

2. Not including your time as a trainee nephrologist (e.g., resident, trainee, registrar), how many years have you been in practice as a nephrologist?

3. Approximately how many ADULT dialysis patients do you currently treat? (UK: Approximately how many ADULT dialysis patients does your unit have [include hospital and satellite units]?

4. Of the [AMOUNT IN Q.S5] dialysis patients you currently treat, how many are treated with each of the following therapies?

US:

	Total Number of Patients
In-center hemodialysis	
Peritoneal dialysis (PD)	
Home hemodialysis (HHD)	
	Total S5 Amount

France:

	Total Number of Patients
In-centre haemodialysis (HDC)	
On-line haemodiafiltration (HDF)	
Unité de dialyse médicalisée (UDM)	
Dialyse peritonéale (DP)	
Home haemodialysis (HHD)	
Autodialyse centre	
	Total S5 Amount

UK:

	Total Number of Patients
In-centre haemodialysis	
On-line haemodiafiltration (HDF)	
Satellite centres	
Peritoneal dialysis (PD)	
Home haemodialysis (HHD)	
	Total S5 Amount

Germany:

	Total Number of Patients
In-center haemodialysis	
On-line haemodiafiltration (HDF)	
Peritoneal dialysis (PD)	
Home haemodialysis (HHD)	
Limited care (self-care at an ICHD clinic	
	Total S5 Amount

Canada:

	Total Number of Patients
In-centre hemodialysis (including self and satellite care)	
On-line hemodiafiltration (HDF)	
Peritoneal dialysis (PD)	
Home hemodialysis (HHD)	
	Total S5 Amount

5.1. You indicated that you do not currently have any patients on home hemodialysis. Does your dialysis clinic (“unit” in UK) offer home hemodialysis?

a) YES

b) NO

5.2. You indicated that you do not currently have any patients on peritoneal dialysis. Does your dialysis clinic (“unit” in UK) offer peritoneal dialysis?

a) YES

b) NO

Physician attitudes

The following statements refer to your attitudes about treating dialysis patients. Please indicate how strongly you AGREE or DISAGREE with each statement using the scale below.

Strongly Disagree	Disagree	Somewhat Disagree	Somewhat Agree	Agree	Strongly Agree
1	2	3	4	5	6

A. Personal motivations for treating chronic kidney disease (or dialysis) patients

1. Improving dialysis patients' quality of life is more important to me than helping them live longer
2. Treating patients with chronic kidney disease is more frustrating than rewarding

B. Treatment goals

3. I wait to start a patient on dialysis until it is absolutely necessary
4. I frequently adjust my patients' dialysis regimens (even in small ways) in order to make sure their therapy is optimal
5. Increasing the frequency of dialysis beyond 3 times per week significantly improves clinical outcomes
6. Longer dialysis sessions done nocturnally would result in significantly better clinical outcomes than traditional in-center dialysis
7. I believe urea clearance is a better measure of dialysis efficacy than middle molecule clearance (e.g., beta-2, phosphate)
8. I will try to persuade my patients to choose the dialysis modality that offers the best clinical outcomes even if they are nervous about trying it

C. Attitudes towards dialysis modalities

Home vs. in-center dialysis

9. I worry that home dialysis patients (PD or HHD) will not comply with their treatment regimen
10. I prefer having my patients do in-center dialysis because it allows me to monitor their condition more closely
11. The interactions that in-center dialysis patients have with each other are a valuable part of helping them cope with their illness
12. In-center dialysis is less stressful for patients than home dialysis

13. I encourage patients to consider home dialysis (PD or HHD) because it gives them the flexibility to dialyze at any time of day or night that is convenient for them
14. The high quality professional care patients receive with in-center dialysis is more important than the flexibility they get with home dialysis (PD or HHD)
15. The ability to travel is extremely important to most dialysis patients
16. I avoid prescribing home dialysis (PD or HHD) because patients do not want to bring their disease into their homes
17. I recommend home dialysis (PD or HHD) only to my healthiest patients
18. Most in-center dialysis patients find it a burden to travel to the clinic three times each week

Peritoneal dialysis

19. I try to put as many patients as possible on PD before going to hemodialysis
20. PD is under-prescribed
21. I avoid prescribing PD because I am concerned about peritonitis
22. PD requires higher physician and staff workload than in-center dialysis
23. PD provides patients with a better quality of life than in-center dialysis
24. Patients on PD have better outcomes than patients on traditional in-center dialysis
25. GERMANY ONLY: I educate all patients on PD without pre-selecting capable patients

Home hemodialysis

26. HHD provides better clinical outcomes than peritoneal dialysis
27. HHD provides patients with a better quality of life than in-center dialysis
28. I would be willing to consider prescribing HHD for a patient whose care partner (i.e., a family member or friend who could assist with treatment if needed) does not live in the same residence
29. The risks to patients of nocturnal HHD outweigh the benefits
30. Treating an HHD patient is much more of a burden for physicians than treating an in-center hemodialysis patient
31. Starting a new HHD program and making it successful is too complex
32. HHD is under-prescribed
33. The risk of infection in HHD is too high
34. With proper training, most patients can learn to do HHD safely and effectively

35. I am not comfortable recommending HHD until I see more clinical data
36. UK: I believe that satellite centres provide the benefits of home dialysis (e.g., convenient location and scheduling flexibility) without the drawbacks (e.g., lack of professional help in an emergency situation)
37. Canada: I believe that satellite centers provides the benefits of home dialysis (e.g., convenient location and scheduling flexibility) without the drawbacks (e.g., lack of professional help in an emergency situation
38. France only: The most important benefit of auto-dialyse is that it helps patients keep their illness out of their home
39. There is convincing clinical data that proves HHD patients have better outcomes than in-center patients
40. Current HHD systems are too complicated
41. I would be willing to invest the time to help start an HHD program at my center/I would help start an HHD program if I switch to a clinic (unit in UK) that did not currently offer HHD
42. Patients must have a care partner in order for me to consider prescribing HHD
43. Nocturnal in-center dialysis is better for patients than nocturnal HHD
44. More frequent dialysis helps patients restore clinical balance as compared to patients who dialyze three times per week
45. Significant improvements in HHD technology must be made before I will recommend HHD to more patients

Patient volumetric

1. Thinking about all of your dialysis patients (UK only: your unit's dialysis patients), approximately what percentage have a committed care partner (i.e., a family member or friend who could assist with a home treatment if needed)? Your best estimate is fine.
   a. Committed care partner who lives in the same home as the patient _____%
   b. Committed care partner who does <u>not</u> live in the same home as the patient _____%
   c. No committed care partner _____%

TOTAL _____%

Total must equal 100%

2. Approximately what percentage of your patients (UK only: your unit's dialysis patients) currently on dialysis started dialysis within the last 12 months? Your best estimate is fine.

_______%

Definitions of healthy states

a. Relatively healthy (for example, patients are stable during dialysis and may have 3 co-morbidities that are well-controlled) Moderately healthy (for example, patients are usually stable during dialysis and may have 3 co-morbidities that are moderately controlled)
b. Chronically unhealthy (for example, some patients may not be stable during dialysis, may have 4 or more co-morbidities, and/or may have co-morbidities that are uncontrolled)

Patient types

For this next section, we are going to show you seven different patient profiles. Please read the patient descriptions carefully and think about your most likely dialysis treatment for each patient. We understand that this may be difficult without an actual consultation with the patient. We only ask for your best estimate based on the information provided.

The seven profiles that you will see will be patients who are either on dialysis or ready to start dialysis. Assume all of these patients are medically suitable, open to home dialysis (PD and home hemodialysis), and capable of performing home dialysis. Also, assume that each patient has a committed family member who could assist with treatment if needed. Characteristics which differ across the patient profiles are listed. Factors not listed (such as age, gender, and ethnicity) could vary within a given profile.

Patient #1

- Currently a CKD 4 patient – planning to transition to dialysis shortly
- Relatively or moderately healthy (i.e., may have up to 3 co-morbidities but they are moderately to well controlled)
- Has at least 750 ml of urine output per day
- Would be open to and capable of performing both PD and home hemodialysis
- Has a committed family member who can assist with treatment if needed

Patient #2	
• Currently a CKD 4 patient – planning to transition to dialysis shortly<br>• Relatively or moderately healthy (i.e., may have up to 3 co-morbidities but they are moderately to well controlled)	• Has minimal urine output<br>• Would be open to and capable of performing home dialysis (PD and HHD)<br>• Has a committed family member who can assist with treatment if needed

Patient #3	
• Patient with a kidney transplant that is no longer working – planning to transition to dialysis shortly<br>• Relatively or moderately healthy (i.e., may have up to 3 co-morbidities but they are moderately to well controlled)	• Would be open to and capable of performing home dialysis (PD and HHD)<br>• Has a committed family member who can assist with treatment if needed

Patient #4	
• Currently on PD but it is no longer effective<br>• A switch to hemodialysis is required	• Would be open to and capable of performing home hemodialysis<br>• Has a committed family member who can assist with treatment if needed

Patient #5	
• Currently on ICHD<br>• Chronically unhealthy (i.e., 4 or more co-morbidities which are frequently uncontrolled)	• Has trouble maintaining dry weight (i.e., requires additional dialysis sessions)<br>• Would be open to and capable of performing home dialysis (PD and HHD)<br>• Has a committed family member who can assist with treatment if needed

Patient #6	
• Currently on ICHD<br>• Moderately healthy (i.e., may have up to 3 co-morbidities but they are moderately controlled)	• Would be open to and capable of performing home dialysis (PD and HHD)<br>• Has a committed family member who can assist with treatment if needed

Patient #7	
• Currently on ICHD<br>• Relatively healthy (i.e., may have up to 3 co-morbidities but they are well controlled)	• Would be open to and capable of performing home dialysis (PD and HHD)<br>• Has a committed family member who can assist with treatment if needed

Patient #8	
• Currently on Autodialyse	• Would be open to and capable of performing home dialysis (PD and HHD)
• Moderately to Relatively healthy (i.e., may have up to 3 co-morbidities but they are moderately to well controlled)	• Has a committed family member who can assist with treatment if needed

PT4. Of your last 100 patients who fit this profile, how would you recommend treating these patients today? If none would receive a modality, enter "0"

a. ICHD _____
b. Currently available HHD systems _____
c. PD _____
d. Autodialyse (FRANCE ONLY) _____

TOTAL _____

Total must equal 100

PT6. For your last 100 patients who fit this profile, what dialysis modality would you expect these patients to be using 1 year from now?

a. Remain on ICHD _____
b. Switch to currently available HHD system _____
c. Switch to PD _____
d. Switch to Autodialyse (FRANCE ONLY) _____

TOTAL _____

Total must equal 100

Please indicate how strongly you agree or disagree that each of the following statements describes your overall satisfaction with [INSERT MODALITY 1] or [INSERT MODALITY 2].

<u>LOYALTY</u>

1. Is my preferred choice for my dialysis patients
2. Is a therapy I would encourage my peers to prescribe for more of their patients
3. Is a therapy I expect to recommend to more patients during the next 3-5 years
4. Is the therapy I would recommend to a close friend or family member if they needed dialysis
5. Is the therapy I trust the most

Demographics

Finally, we would like to ask you a few questions about you and your practice.

1. Please indicate what percentage of your time you spend in each of the settings in which you practice. If you do not spend any time in that setting, please enter 0. (Select all that apply.) (Programmer, these did not add up, appears to not add in the Other)

- ☐ Private practice office (UK: Drop office)
- ☐ Hospital (academic or non-academic) or urgent care (FRANCE: Public Hospital or urgent care)
- ☐ Community health clinic (FRANCE: Association)
- ☐ Nursing home or long-term care facility (FRANCE: Nursing home or elderly care)
- ☐ Other (specify:____________)

THANK YOU FOR YOUR TIME
